# Synthetic Biology and Metabolic Engineering of Microalgae for Sustainable Lipid and Terpenoid Production: An Updated Perspective

**DOI:** 10.1111/pbi.70405

**Published:** 2025-10-21

**Authors:** Ty Shitanaka, Yu Wang, Sally Do, Julia Yuson, Samir Kumar Khanal, Krzysztof Zienkiewicz, Zhi‐Yan Du

**Affiliations:** ^1^ Department of Molecular Biosciences & Bioengineering University of Hawaii at Manoa Honolulu Hawaii USA; ^2^ Department of Civil and Environmental Engineering The Hong Kong University of Science and Technology Kowloon Hong Kong; ^3^ Centre for Modern Interdisciplinary Technologies Nicolaus Copernicus University in Toruń Toruń Poland

**Keywords:** carotenoids, metabolic engineering, microalgae, polyunsaturated fatty acids, (PUFAs) sterols, terpenes

## Abstract

Microalgae are increasingly recognised as powerful platforms for the sustainable production of lipids and terpenoids, with expanding applications in the food, fuel and biomanufacturing industries. In this updated review, we consolidate and critically assess the most recent advances in synthetic biology and metabolic engineering of key microalgal models, including *Chlamydomonas reinhardtii* , *Nannochloropsis* spp. and *Phaeodactylum tricornutum* . We focus on developments that have emerged in the latest waves of research, emphasising novel genetic toolkits that accelerate the Design‐Build‐Test‐Learn (DBTL) cycle, breakthroughs in genome‐scale metabolic modelling, and innovative strategies for organelle‐targeted biosynthesis of high‐value compounds. Recent case studies are compared to highlight trends in successful engineering approaches. By capturing these up‐to‐date insights, this review outlines the current trajectory of microalgal biotechnology toward scalable, carbon‐neutral biofactories for polyunsaturated fatty acids (PUFAs) and diverse terpenoids, reinforcing their role in global sustainability and the circular bioeconomy.

## 1 Current Challenges and Opportunities in Sustainable Production of PUFAs, Terpenes and Sterols via Microalgal Platforms

Polyunsaturated fatty acids (PUFAs), terpenes and sterols are essential, high‐value compounds that are crucial for human health. PUFAs play a critical role in human development, health maintenance and disease prevention, particularly the omega‐3 and omega‐6 fatty acids, such as arachidonic acid (ARA), eicosapentaenoic acid (EPA) and docosahexaenoic acid (DHA) (Crawford et al. 2023; Djuricic and Calder 2023). Terpenes are widely used across the pharmaceutical, food, fragrance and biofuel industries (Einhaus et al. 2021), while sterols provide health benefits such as anti‐inflammatory, cholesterol‐lowering and anticancer activities (Potijun et al. 2021; Randhir et al. 2020). However, current production rates of PUFAs, terpenes and sterols are often too low for commercial viability. For instance, omega‐3 PUFA production still heavily depends on unsustainable fish oil extraction, while the global catch of wild forage fish has plateaued in recent years, driving up fish oil prices amid rising demand (Cottrell et al. 2020; Ghamkhar and Hicks 2020). This challenge is further compounded by climate change, which could reduce DHA levels in coastal regions by 10%–58% (Colombo et al. 2020), and by similarly pressing issues in terpene production, where low yields and high costs remain major limitations (Einhaus et al. 2021). Moreover, land‐based sources of sterols also compete with food crops for arable land, raising concerns about long‐term sustainability (Randhir et al. 2020). All these limitations highlight the need for new strategies that enable high‐yield, cost‐effective production of these compounds.

Microalgae have long been recognised as promising and sustainable sources of high‐value compounds due to their rapid growth, high photosynthetic efficiency and remarkable capacity to synthesise diverse secondary metabolites (Einhaus et al. 2021; Huesemann et al. 2023; Randhir et al. 2020; Vecchi et al. 2020; Xu 2022). To fully harness this potential and address challenges such as low natural yields and high production costs, metabolic engineering has emerged as a transformative approach. The last two decades of research have indeed showed that modulation of key genes, optimisation of precursor supply and redirection of carbon flux have proven effective in enhancing the accumulation of specific target compounds in microalgae. Nevertheless, compared to model organisms like *Escherichia coli* and *Saccharomyces cerevisiae* , metabolic engineering in microalgae remains in its infancy, hindered by limited genetic tools, transformation protocols and standardised workflows.

**FIGURE 1:**
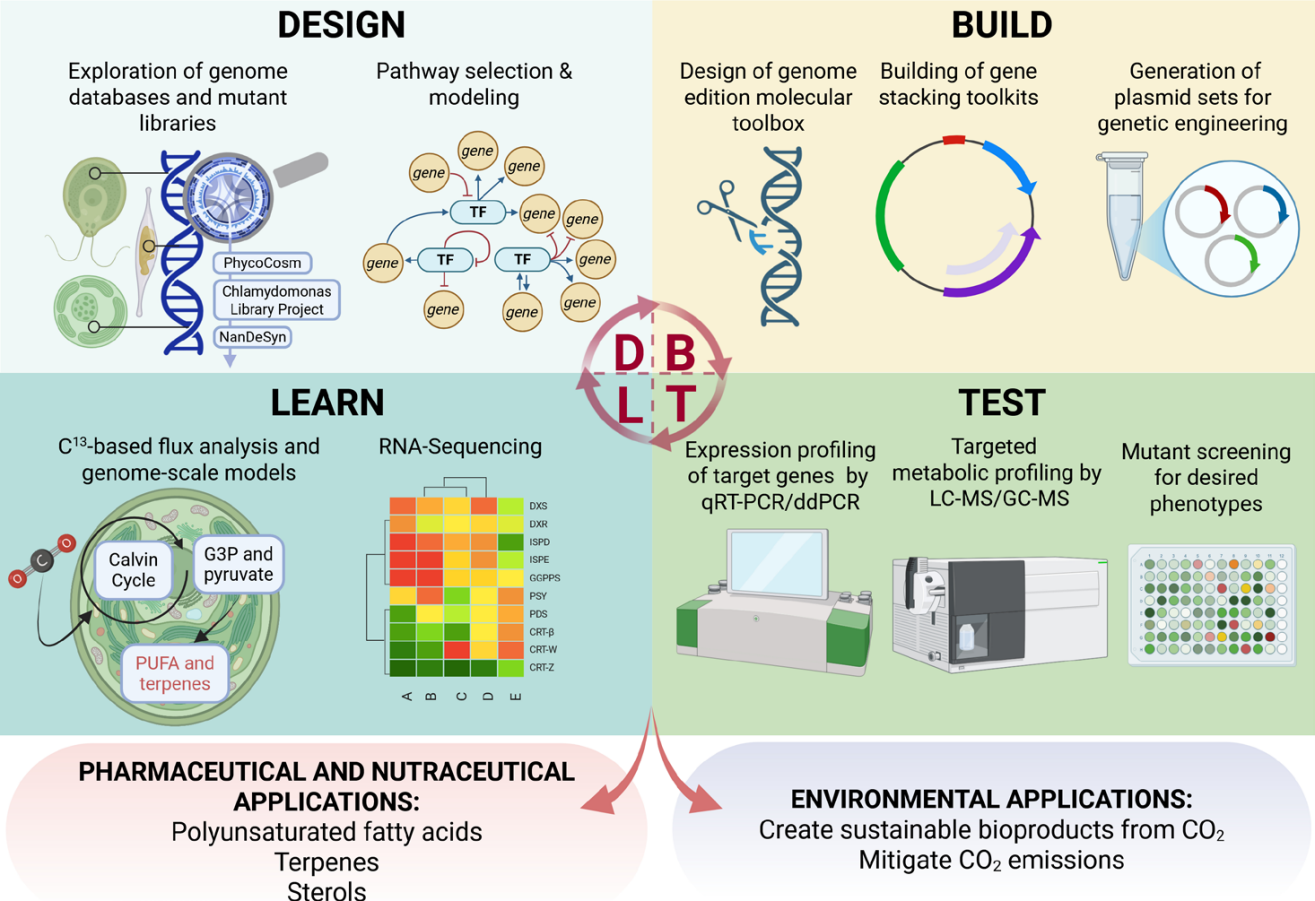
The Design‐Build‐Test‐Learn (DBTL) Cycle for microalgae metabolic engineering. The DBTL systematic approach used in the metabolic engineering of microalgae to optimise bioproduct synthesis consists of four key phases: (1) Design, which involves exploring genome databases and mutant libraries (e.g., *PhycoCosm*, *Chlamydomonas Library Project*, *NanDeSyn*) to identify target pathways. Computational modelling is used for pathway selection and transcriptional regulation analysis; (2) Build, oriented towards construction and development of genome‐editing molecular tools, gene‐stacking toolkits and plasmid sets for genetic engineering, enabling precise modifications of the target organism; (3) Test, where engineered strains undergo validation through targeted metabolic profiling (e.g., LC–MS, GC–MS) and expression analysis of key genes using qRT‐PCR or dPCR. Mutant strains are screened for desired phenotypes to assess improvements in metabolic performance; (4) Learn, where experimental data are integrated to refine metabolic models. Methods such as ^13^C‐based flux analysis, genome‐scale modelling and RNA sequencing help evaluate metabolic shifts and optimise pathways for enhanced bioproduct yield. The DBTL cycle accelerates the development of microalgae as sustainable biofactories for pharmaceutical and nutraceutical applications (e.g., polyunsaturated fatty acids, terpenes, sterols) and environmental applications, including carbon capture and sustainable bioproduct generation.

Although nearly a decade has passed since the Design‐Build‐Test‐Learn (DBTL) framework was introduced into microalgal molecular engineering, it is clear that it has significantly accelerated the rational development of engineered microalgal strains (Figure 1, Table 1). This iterative approach integrates computational design, genetic toolkits, quantitative testing and model‐based learning to optimise specific metabolic pathways. This review focuses on these tools and strategies used in the metabolic engineering of microalgae, with an emphasis on engineering approaches that have demonstrated at least 1‐fold increases in target product titres, which is our chosen threshold for functional relevance and biological significance. Rather than reviewing general biosynthetic pathways that can be found elsewhere, we assess the strategies that underpin recent engineering successes. We structured this review around three major model microalgal species, *Chlamydomonas reinhardtii* , *Nannochloropsis* spp. and *Phaeodactylum tricornutum* , which are currently the most advanced in terms of tool development, transformation efficiency and functional validation. These species represent green algae (chlorophytes), eustigmatophytes and diatoms, respectively, and offer unique physiological and genetic contexts for metabolic engineering. Their diversity allows for a meaningful comparison of tool effectiveness and strain performance. While other species are mentioned throughout, including extremophilic microalgae and cyanobacteria, we focus on the three model species as they are the best characterised, with the most extensive genetic tools and data available.

**TABLE 1:** Key tools, databases and engineering platforms used in each phase of the Design‐Build‐Test‐Learn (DBTL) cycle for microalgal metabolic engineering.

DBTL phase	Tool/Database/Toolkit	Species	Function/Description	References
Design	PhycoCosm	Various algae	Comparative genomics and multi‐omics database	Grigoriev et al. (2021)
	Plant metabolic network (PMN v15)	Plants & algae	Curated metabolic pathways, including lipids and terpenes	Hawkins et al. (2021)
	Chlamydomonas library project (CLiP)	*C. reinhardtii*	~60,000 insertional mutants	Li et al. (2019); Wang et al. (2023)
	NanDeSyn	*Nannochloropsis* spp.	Transcriptomic, proteomic and gene annotation data	Gong et al. (2020)
Build	MoClo toolkit	*C. reinhardtii*	Modular cloning system for gene stacking	Crozet et al. (2018)
	pOpt3 plasmid set	*C. reinhardtii*	Fluorescent reporters, chloroplast targeting peptides	Gutiérrez et al. (2022)
	CRISPR/Cas9 & Cas12a	*Nannochloropsis*	Marker‐free genome editing; CRISPRi tools	Naduthodi et al. (2021)
	Universal loop assembly	*P. tricornutum*	Decoupled plasmid and transcription unit system	Pollak et al. (2020)
	RICE & EE systems	*P. tricornutum*	Chromosomal vs. episomal expression comparison	George et al. (2020)
Test	LC–MS/GC–MS	All species	Quantitative metabolite profiling	—
	qRT‐PCR/ddPCR	All species	Gene expression analysis	—
	FACS	*C. reinhardtii*	High‐throughput phenotypic screening	Sproles et al. (2022)
Learn	GEMs, pcGEMs	*C. reinhardtii* , *Nannochloropsis*	Genome‐scale and protein‐constrained models	Arend et al. (2023)
	^13^C‐metabolic flux analysis	All species	Carbon flow mapping and optimisation	Volk et al. (2023)

Overall, here we present a comprehensive, method‐focused synthesis of recent advances in microalgal metabolic engineering for PUFAs, terpenes and sterols production. By framing the review within the DBTL cycle and highlighting impactful case studies and enabling technologies, we aim to provide up‐to‐date insights into the tools and strategies driving high‐yield bioproduct synthesis in engineered microalgal strains.

## 2 Synthetic Biology and the DBTL Cycle: Accelerating Rational Design of Algal Cell Factories

As mentioned above, the application of synthetic biology to microalgae has been significantly enhanced through the use of iterative metabolic engineering frameworks like the DBTL cycle (Figure 1). Consequently, this systematic approach provides a robust methodology for improving the metabolic output of target molecules such as PUFAs, carotenoids, terpenes and sterols. By cycling through computational design, genetic construction, phenotypic testing and data‐driven learning, it is possible to optimise production strains with increased precision and efficiency. To facilitate systematic strain engineering, a growing toolbox now supports each phase of the DBTL cycle in microalgae. These include genome‐editing technologies, modular cloning systems, high‐throughput screening platforms and computational modeling resources. Table 1 summarises representative tools and resources that have been effectively applied to advance microalgal metabolic engineering within the DBTL framework. Their key characteristics and applications are detailed in the text below.

In the **design phase**, bioinformatic tools and multi‐omics datasets are leveraged to identify key metabolic bottlenecks, regulatory nodes and potential engineering targets. Databases such as *PhycoCosm* (www.phycocosm.jgi.doe.gov) or the *Plant Metabolic Network* (PMN, www.plantcyc.org) facilitate genome‐scale comparisons and gene function annotations across various algal species (Grigoriev et al. 2021; Hawkins et al. 2021). Recent advances in systems biology, including genome‐scale metabolic models and flux balance analysis (FBA), have also started to support rational metabolic target identification in microalgal systems. Additionally, resources like the *Chlamydomonas Library Project* CLiP, (www.chlamylibrary.org) offer high‐throughput mutant libraries that help functionally validate gene candidates before engineering interventions (Li et al. 2019; Wang et al. 2023). The **build phase** employs synthetic biology tools to construct and introduce genetic elements for pathway rewiring. Modular cloning systems such as MoClo (Crozet et al. 2018), Loop assembly, and Golden Gate enable efficient multigene construct assembly. In *C. reinhardtii* , the pOpt3 expression vectors facilitate targeted nuclear expression, often linked to organelle‐targeting sequences for precise subcellular localisation (Gutiérrez et al. 2022). CRISPR/Cas9 genome editing has been successfully applied in *Nannochloropsis oceanica* for targeted gene knockouts and promoter engineering, using both plasmid‐based delivery and ribonucleoprotein (RNP) complexes (Naduthodi et al. 2021; Poliner, Takeuchi, et al. 2018). In *P. tricornutum* , metabolic engineering efforts have leveraged two distinct expression strategies: randomly integrated chromosomal expression (RICE) and extrachromosomal episomal vectors. RICE lines displayed greater variability in transgene expression, with higher maximum levels but lower baseline stability, while episomal systems provided more consistent expression profiles. It has been proposed that the most productive RICE clones may have integrated transgenes into genomic safe harbour loci, enhancing expression (George et al. 2020). The **test phase** involves evaluating engineered strains using biochemical, transcriptomic and physiological assays. Analytical platforms such as gas chromatography–mass spectrometry (GC–MS), liquid chromatography‐mass spectrometry (LC–MS) and nuclear magnetic resonance (NMR) are widely used to quantify metabolite production. Simultaneously, gene expression can be validated via RT‐qPCR, droplet digital PCR (ddPCR), or fluorescent reporter systems. These measurements help to confirm both the efficacy and stability of the genetic modifications while also revealing potential trade‐offs in growth or photosynthetic performance. Finally, the **learn phase** integrates experimental data into updated models to guide the next iteration of the DBTL cycle. Constraint‐based modelling techniques and proteome‐informed FBA approaches have been developed for *C. reinhardtii* and related strains to simulate cellular responses to genetic perturbations (Arend et al. 2023). Machine learning methods are also beginning to be incorporated to predict construct success rates and optimise metabolic fluxes. This iterative feedback loop enhances engineering efficiency and improves product yields in subsequent cycles.

Summing up, by adopting the DBTL approach, it is possible to systematically identify the most effective strategies for enhancing the production of target biomolecules. In the following chapters, we examine how the DBTL framework has been applied to improve the biosynthesis of PUFAs, terpenes and sterols in microalgae. Emphasis is placed on engineering studies that report at least twofold increases in product titres, as these represent significant progress in the context of strain development and industrial scalability.

## 3 Metabolic Strategies to Enhance Sustainable PUFA Production in Microalgae

PUFAs are essential biomolecules whose accumulation in microalgae is influenced by various environmental factors such as nutrient availability and light intensity (Zakaria et al. 2025). However, while external conditions modulate PUFA levels, the low inherent yields of target PUFAs in native strains have prompted an increasing focus on genetic and metabolic engineering approaches to boost biosynthetic capacity. Current research efforts prioritise identifying and manipulating key genes involved in PUFA biosynthesis, either by overexpression or knockout, to increase the flux of carbon toward ARA, EPA and DHA. This section presents an integrated overview of PUFA biosynthetic pathways, followed by a detailed assessment of the genetic engineering strategies that have demonstrably enhanced PUFA production in various microalgal species.

### 3.1 Overview of PUFA Biosynthesis in Microalgae

**FIGURE 2:**
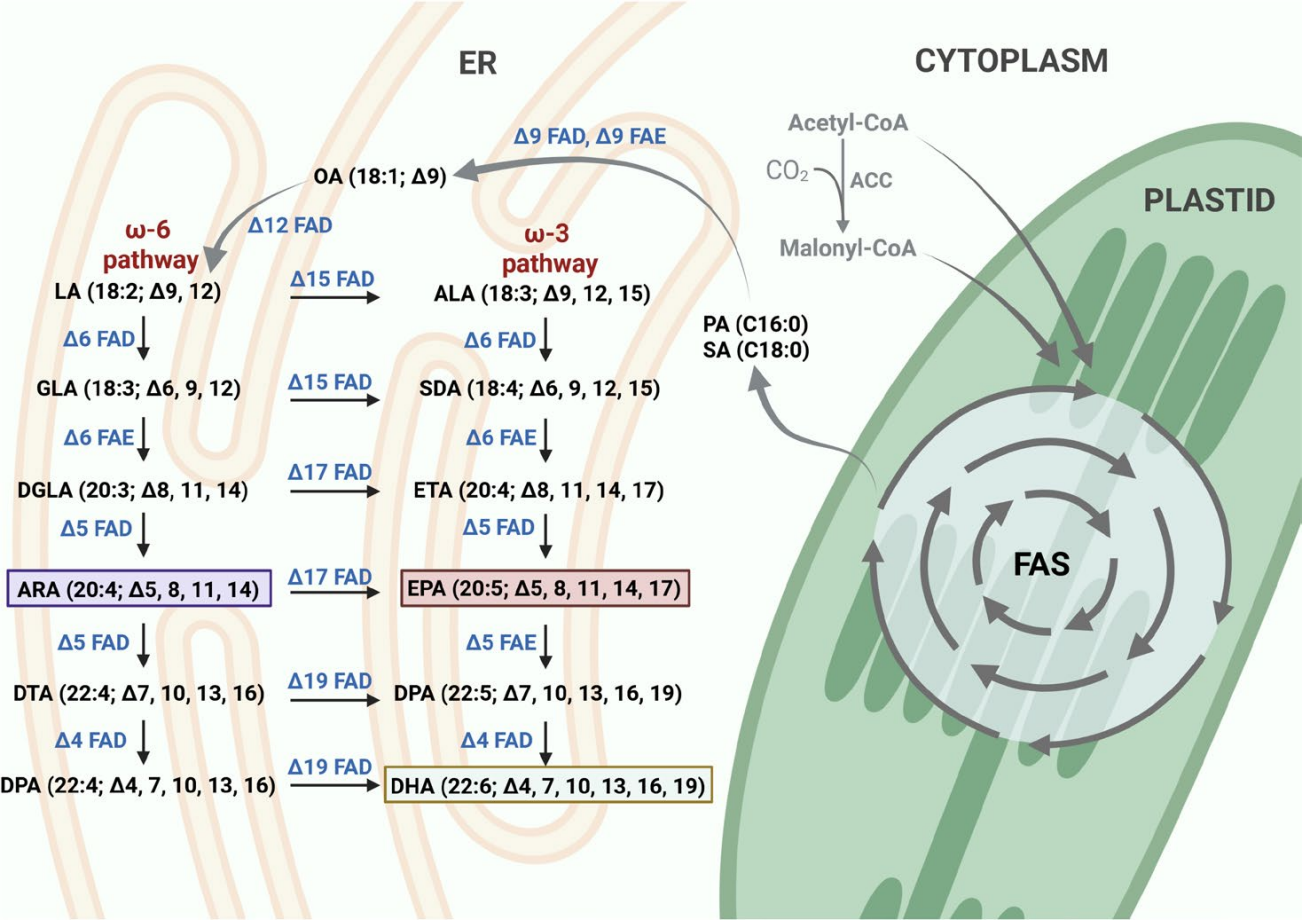
Metabolic pathways involved in the biosynthesis of omega‐6 (*ω*‐6) and omega‐3 (*ω*‐3) polyunsaturated fatty acids (PUFAs) in microalgae. The biosynthesis begins in the plastid, where acetyl‐CoA is converted into malonyl‐CoA by acetyl‐CoA carboxylase (ACC), leading to the production of saturated fatty acids such as palmitic acid (PA, C16:0) and stearic acid (SA, C18:0) through fatty acid synthesis (FAS). These fatty acids are then transported to the ER for further modifications, including desaturation and elongation. In the ER, desaturation and elongation enzymes catalyse the conversion of precursor fatty acids into bioactive long‐chain polyunsaturated fatty acids (LC‐PUFAs). The *ω*‐6 pathway, indicated in red, starts with linoleic acid (LA, 18:2), which undergoes a series of desaturation and elongation reactions to ultimately produce arachidonic acid (ARA, 20:4). The *ω*‐3 pathway, also in red, begins with α‐linolenic acid (ALA, 18:3), which follows a similar set of reactions leading to the synthesis of eicosapentaenoic acid (EPA, 20:5) and docosahexaenoic acid (DHA, 22:6). Several key enzymes facilitate these conversions. Δ6 desaturase (Δ6 FAD) introduces a double bond, converting LA to γ‐linolenic acid (GLA) and ALA to stearidonic acid (SDA). Δ5 desaturase (Δ5 FAD) further processes dihomo‐γ‐linolenic acid (DGLA) to ARA and eicosatetraenoic acid (ETA) to EPA. Elongases (EL) extend the carbon chain length of fatty acids, while Δ4 desaturase (Δ4 FAD) catalyses the final conversion of docosapentaenoic acid (DPA) to DHA. The final products of the *ω*‐6 pathway include ARA, an essential precursor for eicosanoids, while the *ω*‐3 pathway leads to the production of EPA and DHA.

The biosynthesis of PUFAs in microalgae has been well‐characterised in numerous literature sources. Therefore, here we briefly summarise the core pathways to provide the necessary context for subsequent engineering strategies, focusing on the key enzymatic steps relevant to current metabolic interventions (Figure 2). PUFA biosynthesis involves a series of elongation and desaturation steps facilitated by elongases and desaturases, respectively. Elongases extend fatty acid chains through the condensation of acyl‐CoA with malonyl‐CoA, while desaturases introduce cis double bonds at specific positions along the acyl chain. The biosynthesis initiates in the plastid with acetyl‐CoA, which is produced via glycolysis and TCA cycle metabolism. Acetyl‐CoA is first converted to malonyl‐CoA by acetyl‐CoA carboxylase (ACC), marking the committed step in fatty acid synthesis. In the chloroplast, malonyl‐CoA is converted to malonyl‐ACP by malonyl‐CoA:ACP transacylase (MCAT), which then enters the fatty acid synthase (FAS) complex to generate saturated fatty acids such as palmitic acid (C16:0) and stearic acid (C18:0). These acyl groups are subsequently desaturated or elongated by plastid or ER‐localised enzymes. Oleic acid (OA; C18:1, *ω*‐9), produced from stearoyl‐ACP, serves as a key branching point. It is desaturated to linoleic acid (LA; C18:2, *ω*‐6) via a Δ12 desaturase, initiating either the *ω*‐6 or *ω*‐3 PUFA pathways. The *ω*‐6 pathway converts LA into γ‐linolenic acid (GLA; C18:3), then to dihomo‐γ‐linolenic acid (DGLA; C20:3), and finally to ARA (C20:4) through successive elongation and desaturation steps. Alternatively, LA can be converted to α‐linolenic acid (ALA; C18:3, *ω*‐3) by Δ15 desaturase, which then follows a similar route to yield EPA (C20:5) and DHA (C22:6). Δ6, Δ5 and Δ4 desaturases, along with elongases such as Δ6 and Δ5 elongases, play critical roles in modulating flux through these branches (Figure 2).

### 3.2 Engineering Approaches for Target PUFAs Enrichment in Microalgal Systems

Microalgae are valuable platforms for PUFA biosynthesis owing to their native ability to accumulate *ω*−3 and *ω*−6 fatty acids. Table 2 summarises the most recent engineering studies in which the overexpression or knockdown of specific desaturases and elongases has resulted in enhanced PUFA accumulation.

**TABLE 2:** Metabolic engineering of PUFA production in microalgae.

Products	Species	Target gene/Strategy	Production	References
ARA	*N. oceanica*	Overexpression of NoΔ6FAD	~2.0‐fold increase in ARA	Yang et al. (2019)
	*L. incisa*	RNAi knockdown of LiΔ6FAE, LiΔ5FAD, LiΔ6FAD, LiΔ12FAD	Lower ARA production in mutants	Kugler et al. (2019)
EPA	*N. oceanica*	Overexpression of NoΔ6FAD	~1.5‐fold increase in EPA	Yang et al. (2019)
	*N. oceanica*	Overexpression of NoΔ9FAD + NoΔ12FAD; NoΔ5FAD + NoΔ9FAD + NoΔ12FAD	1.25‐fold increase in EPA	Poliner, Pulman, et al. (2018)
	*N. oceanica*	Synergistic genetic module engineering (Δ12D, Δ6D, Δ6E, Δ5D, *ω*3D co‐expression)	Up to 34.96% increase in EPA (N replete conditions); 40.47% increase in TAG‐EPA (N deficient conditions)	Zheng et al. (2025)
	*N. oceanica*	Engineering acyl‐ACP thioesterases (PKS/FAS modulation)	Designer lipid production	Wang et al. (2021
	*P. tricornutum*	Knockout of PtΔ9FAD	~1.3–1.4‐fold increase in EPA	Smith et al. (2021)
	*P. tricornutum*	Overexpression of malic enzyme (ME) + PtΔ5FAD	1.5‐fold increase in EPA	Zou et al. (2019)
	*D. salina*	Overexpression of TpFADS6	13.3‐fold increase in EPA	Shi et al. (2018)
	*C. vulgaris*	Starch‐deficient mutant (mutagenesis + ALE + acetic acid)	485% increase in EPA	Zhang et al. (2025)
	*S. limacinum*	Reassembly of the incomplete heterologous FAS pathway for *de novo synthesis*	Achieved EPA biosynthesis from the FAS pathway	Duan et al. (2025)
	*P. tricornutum*	Heterologous co‐expression of Δ6‐ and Δ5‐desaturases + LPCAT from *P. tricornutum* in *Nicotiana tabacum*	Achieved EPA at 4.5% of total FAs in TAGs in *N. tabacum* leaves	Klińska‐Bąchor et al. (2024)
DHA	*N. oceanica*	Overexpression of NoΔ6FAD	2.3‐fold increase in DHA	Yang et al. (2019)
	*P. tricornutum*	Overexpression of PtDGAT2B + OtFAE5	4.7‐fold increase in DHA	Haslam et al. (2020)
	*Schizochytrium* sp.	Overexpression of GGPPS + fermentation optimisation	DHA content up to 13.4 g/L with increased carotenoid yield	Zhang et al. (2024)
	*C. vulgaris*	Starch‐deficient mutant under ALE + acetic acid	161% increase in DHA	Zhang et al. (2025)
	*S. limacinum*	CRISPR/Cas9‐based push‐pull‐block PKS pathway strengthening (ΔPEX10‐ACC1‐DGAT strain)	Lipid content increased up to 77.14%, DHA increased up to 55.10% and PUFA increased up to 70.47%	Duan et al. (2025)

Abbreviations: ACC, acetyl‐CoA Carboxylase; ALE, adaptive evolution strategies; DGAT, diacylglycerol acyltransferase; FAD, fatty acid desaturase; FAE, fatty acid elongase; GGPPS, geranylgeranyl diphosphate synthase; GPAT, glycerol‐3‐phosphate acyltransferase; LPCAT, lysophosphatidic acid acyltransferase; MCAT, malonyl‐CoA‐acyl carrier protein transacylase; ME, malic enzyme; PEX10, peroxisomal E3 ubiquitin ligase; PKS; polyketide synthase; TAG, triacylglycerol.

While the field of ARA engineering is still emerging, a few recent promising outcomes have been achieved. For example, overexpression of the endogenous Δ6 FA desaturase in *N. oceanica* resulted in a 2.0‐fold increase in ARA production (Yang et al. 2019). These results were also corroborated by RNAi‐mediated knockdown experiments in another green microalga *Lobosphaera incisa*, where suppression of Δ6 elongase and Δ5, Δ6 and Δ12 desaturases caused marked reductions in ARA accumulation (Kugler et al. 2019), suggesting these enzymes are critical control points in the ARA biosynthetic pathway. To further advance ARA production, future research should also prioritise non‐model microalgal species that naturally accumulate high levels of ARA, such as *Porphyridium purpureum* (Li et al. 2020) or *Myrmecia incisa* (Guo et al. 2022). Stepwise reconstruction of the ARA pathway in model systems like *C. reinhardtii* or *P. tricornutum* may also help identify rate‐limiting steps and allow fine‐tuned optimisation. On the other hand, comparative kinetic analyses of Δ5 desaturases across diverse microalgal species could lead to the discovery of highly efficient variants suitable for metabolic PUFA engineering.

EPA is the most frequently targeted PUFA in microalgal engineering due to its commercial value. Overexpression of Δ5 and Δ6 desaturases in, respectively, *P. tricornutum* and *N. oceanica* has yielded EPA titre increases ranging from 1.2 to 1.5‐fold (Yang et al. 2019; Zou et al. 2019) (Table 2). In turn, the heterologous expression of Δ6 desaturase from *Thalassiosira pseudonana* in *Dunaliella salina* led to a 13.3‐fold increase in EPA levels (Shi et al. 2018), representing one of the highest improvements reported. Other effective approaches include combinatorial overexpression of desaturases (Δ12 + Δ9 + Δ5) in *N. oceanica* , though results showed only modest additive effects (Poliner, Pulman, et al. 2018). Targeting competing pathways has also proven effective. Knockdown of Δ9 desaturase in *P. tricornutum* redirected carbon flux away from prokaryotic membrane lipid synthesis toward long‐chain PUFA biosynthesis, resulting in a 1.4‐fold increase in EPA levels (Smith et al. 2021).

Recent studies have extended these strategies. In *N. oceanica* , functional characterisation of a chloroplast–endoplasmic reticulum localised *ω*3‐fatty acid desaturase (No*ω*3‐FAD) confirmed its essential role as the final enzyme in EPA biosynthesis, and its overexpression boosted EPA production to 292 mg/L (Zheng et al. 2025). Similarly, manipulation of chain‐length–specific thioesterases in *N. oceanica* enabled fine‐tuning of PUFA chain‐length distribution, offering a foundation for “designer lipid” production (Wang et al. 2021). Beyond *Nannochloropsis*, a starch‐deficient mutant of *Chlorella vulgaris* generated through chemical mutagenesis and adaptive laboratory evolution under acetic acid supplementation yielded a 485% increase in EPA and 161% in DHA (Zhang et al. 2025). Notably, recent plant‐based engineering approaches have shown that co‐expression of microalgal *ω*3‐pathway desaturases with acyl‐CoA:lysophosphatidylcholine acyltransferase (LPCAT) alleviates the substrate dichotomy bottleneck, increasing the proportion of EPA incorporated into storage lipids (Klińska‐Bąchor et al. 2024).

DHA production is less common in microalgae compared to EPA, but targeted engineering has produced impressive outcomes (Table 2). Overexpression of Δ6 desaturase in *N. oceanica* resulted in a 2.3‐fold increase in DHA (Yang et al. 2019). In turn, in *P. tricornutum* , the introduction of a Δ5 elongase from *Ostreococcus tauri* yielded an eight‐fold increase in DHA, and co‐expression with the native triacylglycerol (TAG)‐producing DGAT2B gene led to a 4.7‐fold increase (Haslam et al. 2020). More recently, CRISPR/Cas9 engineering in *Schizochytrium limacinum* has enabled simultaneous reconstruction of both FAS and polyketide synthase (PKS) pathways, leading to co‐production of EPA and DHA at elevated titers (Duan et al. 2025). Complementary strategies integrating fermentation optimisation with genetic engineering in *Schizochytrium* sp. further enabled co‐production of carotenoids and DHA, reaching high β‐carotene titres while sustaining DHA levels above 13 g/L (Zhang et al. 2024).

All these results highlight the importance of combining metabolic pathway rewiring with culture optimisation to maximise PUFAs productivity in target microalgae. Future work on ARA and DHA engineering may leverage lessons from EPA‐focused strategies, while also exploiting dual‐pathway hosts, such as *Schizochytrium*, that naturally harbour both FAS and PKS systems.

## 4 Terpenoid and Sterol Biosynthesis and Engineering

While advances in PUFA engineering boost the potential of microalgae as high‐value lipid biofactories, recent progress has also been achieved in terpenoid and sterol biosynthesis (Tables 3 and 4). These pathways broaden the product spectrum while introducing distinct challenges and opportunities for metabolic design. To enhance the production of carotenoids, terpenes and sterols, the explored strategies range from random mutagenesis to targeted metabolic engineering. However, although random mutagenesis can disrupt competing pathways or elevate the expression of rate‐limiting enzymes, its imprecision limits broader application. By contrast, metabolic engineering enables precise redirection of carbon flux toward desired products. In the following sections, we review the biosynthesis and engineering of terpenes, carotenoids and sterols, with particular emphasis on studies employing synthetic biology tools that achieved substantial improvements in yield.

**TABLE 3:** Recent studies on metabolic engineering for the production of carotenoids in diverse microalgae.

Products	Species	Strategy/Target gene(s)	Production	References
α‐carotene	*C. reinhardtii*	Overexpression of the wild‐type *ORANGE* (Cr*ORWT*) gene	2.5‐fold increase	Yazdani et al. (2021)
		Overexpression of the mutated *ORANGE* (Cr*ORHis*) gene	3.7‐fold increase	
β‐carotene	*N. oceanica*	Overexpression of endogenous *LCYB* (No*LCYB*)	39% or 49% increase	Liu et al. (2022)
β‐carotene	*D. salina*	CRISPR/Cas9‐mediated knockout of endogenous *BHY* (Dschyb)	Up to 2.2‐fold increase	Hu et al. (2021)
β‐carotene	*C. reinhardtii*	Heterologous expression of *PBS* from *X. dendrorhous* harbouring *PSY* and *LCYB* activities	38%–72% increase	Rathod et al. (2020)
β‐carotene	*C. reinhardtii*	Overexpression of the wild‐type *ORANGE* (Cr*ORWT*) gene	Up to 2.2‐fold increase	Yazdani et al. (2021)
		Overexpression of the mutated *ORANGE* (Cr*ORHis*) gene	3.1‐fold increase	
β‐carotene	*C. reinhardtii*	Heterologous expression of *LCYB* (*CrtY*) from *P. agglomerans*	2.45‐fold increase	Huang et al. (2023)
β‐carotene	*P. tricornutum*	Overexpression of endogenous *VDE*, *VDR* and *ZEP*	Up to 2.4‐fold increase^a^	Manfellotto et al. (2020)
β‐carotene and fucoxanthin	*P. tricornutum*	Co‐overexpression of endogenous *DXS* and *LYCB*	Up to 1.1‐fold in β‐carotene and 0.75‐fold fucoxanthin	Cen et al. (2022)
Cantaxanthin (plus EPA)	*N. oceanica*	Heterologous expression of *BKT* from *C. reinhardtii* (*CrBKT*)	Increase up to 3.3 mg/g (with higher EPA levels)	Liu et al. (2024)
Carotenoids and ketocarotenoids			Increased pools of carotenoids and ketocarotenoids per cell up to 1.5 and 10‐fold, respectively	Canini et al. (2024)
Zeaxanthin	*C. reinhardtii*	CRISPR/Cas9‐mediated *LCYE* + *ZEP* knockout	60% yield increase (up to 5.24 mg/L and 7.28 mg/g DW), further increased to 6.84 mg/L with medium optimisation	Song et al. (2020)
Zeaxanthin	*C. reinhardtii*	CRISPR/Cas9—mediated knockout of *LCYE* and *LCYE* + *ZEP* with overexpression of endogenous *BKH* (*CHYB*)	190‐fold increase with medium modification	Jang et al. (2025)
Lutein	*C. reinhardtii*	Heterologous expression of *PBS* from *X. dendrorhous* harbouring *PSY* and *LCYB* activities	60%–83% increase	Rathod et al. (2020)
Lutein	*C. reinhardtii*	Overexpression of the wild‐type *ORANGE* (Cr*ORWT*) gene	2.3‐fold increase	Yazdani et al. (2021)
		Overexpression of the mutated *ORANGE* (Cr*ORHis*) gene	2.8‐fold increase	
Astaxanthin	*Synechocystis* sp. PCC6803	Codon‐optimised heterologous expression of *BKT* and carotenoid hydroxylase (*crtR‐B*) from *H. pluvialis*	Increase up to ~4.8 mg/g DW	Liu et al. (2019)
Astaxanthin	*H. pluvialis*	Chloroplast transformation with plastid codon‐optimised endogenous *PDS*	Increase by up to 67% per DW and up to 90% per culture volume	Galarza et al. (2018)
Astaxanthin	*C. reinhardtii*	Synthetic redesign and intragenic revival of endogenous but poorly expressed *BKT* (Cr*BKT*)	Up to 50% of total carotenoids converted to astaxanthin, productivity up to 2.5 mg/L/day	Perozeni et al. (2020)
Astaxanthin	*C. reinhardtii*	CRISPR/Cas9‐generated double knockout of *LCYE* and *ZEP*, combined with overexpression of synthetic, codon‐optimised *BKT* (CrBKT)	Accumulation of ketocarotenoids by up to~60%–104%. Up to 71% of total carotenoids converted to astaxanthin/canthaxanthin (75.8% astaxanthin)	Perozeni et al. (2025)
Astaxanthin	*C. reinhardtii*	CRISPR/Cas9‐mediated knockout of *LCYE* combined with co‐overexpression of endogenous *BKT* (Cr*BKT*), PS (*PacrtB*) and *BKH* (Cr*CHYB*)	2.3‐fold increase (1.8 mg/L)	Kneip et al. (2024)
Astaxanthin	*C. reinhardtii*	Synthetic redesign and overexpression of *BKT* (Cr*BKT*), PS (*PacrtB*) and BKH (Cr*CHYB*)	Single *BKT* transformants: increase up to 0.81 mg/L, single Cr*CHYB* tranformants: increase up to 6.2‐fold, triple transformants (*BKT* + *PacrtB* + *CHYB*): increase up to 9.5 mg/L under mixotrophy and up to 23.5 mg/L at high cell density with CO_2_ and high light treatment	Amendola et al. (2023)
Diadinoxanthin	*P. tricornutum*	Overexpression of endogenous *VDE*, *VDR* and *ZEP*	Up to 3.3‐fold increase^a^	Manfellotto et al. (2020)
Diatoxanthin			Up to 3.5‐fold increase^a^	
Fucoxanthin		Overexpression of endogenous *VDE*, *VDR* and *ZEP*	Up to 4.4‐fold increase^a^	
Violaxanthin	*C. reinhardtii*	Overexpression of wild‐type *ORANGE* (Cr*ORWT*) gene	Up to 2.3‐fold increase	Yazdani et al. (2021)
Violaxanthin		Overexpression of mutated *ORANGE* (Cr*ORHis*) gene	Up to 3.4‐fold increase	

a Data estimated from the graph.

**TABLE 4:** Studies on metabolic engineering for the production of terpenes and sterols in diverse microalgae.

Products	Species	Target gene(s)/Strategy	Production	References
Squalene	*P. tricornutum*	Overexpression of endogenous *HMGR* (PtHMGR), its truncated version (*tHMGR*) and heterologous expression of *SQE* from *N. oceanica* (No*SQE*)	Up to 10‐fold increase (*HMGR*); up to 4‐fold increase for tHMGR and no difference for *NoSQE*‐expressing lines	Jaramillo‐Madrid et al. (2020)
	*Schizochytrium* sp.	Overexpression of endogenous acetyl‐CoA C‐acetyltransferase (*ACAT*) (combined with Fe starvation)	86% increase	Xu et al. (2023)
	*Schizochytrium* sp.	Endogenous promoter optimisation for *SQS*	Up to 2.8‐fold increase	Nong et al. (2024)
Cycloartenol	*P. tricornutum*	Over‐expression of endogenous *HMGR* (PtHMGR), its truncated version (*tHMGR*) and heterologous expression of *SQE* from *N. oceanica* (No*SQE*)	Up to 3‐fold increase for HMGR; ~1.8‐fold increase for tHMGR; ~1.8‐fold increase for NoSQE‐expressing lines	Jaramillo‐Madrid et al. (2020)
Obtusifoliol	*P. tricornutum*	Overexpression of endogenous *HMGR* (PtHMGR), its truncated version (*tHMGR*) and heterologous expression of *SQE* from *N. oceanica* (No*SQE*)	Up to 2.5‐fold increase for HMGR; ~2.0‐fold increase for tHMGR; ~1.8‐fold increase for No*SQE*‐expressing lines	Jaramillo‐Madrid et al. (2020
Campesterol	*P. tricornutum*	Overexpression of endogenous *HMGR* (PtHMGR), its truncated version (*tHMGR*) and heterologous expression of *SQE* from *N. oceanica* (No*SQE*)	Up to ~2‐fold increase for PtHMGR and ~3‐fold increase for No*SQE*‐expressing lines	Jaramillo‐Madrid et al. (2020)
24‐methylenecholesta‐5,24 (24′)‐dien‐3β‐ol	*P. tricornutum*	Overexpression of endogenous *HMGR* (PtHMGR), its truncated version (*tHMGR*) and heterologous expression of *SQE* from *N. oceanica* (No*SQE*)	Up to 17‐fold increase for HMGR; ~10‐fold increase for tHMGR; ~10‐fold (normally absent in WT)	Jaramillo‐Madrid et al. (2020)
Lupeol	*P. tricornutum*	Heterologous expression of *Lotus japonicus* lupeol synthase (LS)	Increase up to ~0.1 mg/g DW	D'Adamo et al. (2019)
Betulinic acid	*P. tricornutum*	Heterologous co‐expression of *Medicago truncatula* Cytochrome P450 (CYP716A12) and its native reductase (CPR)	Increase up to 0.1 mg/g DW	D'Adamo et al. (2019)
Casbene	*N. oceanica*	Heterologous expression of *Daphne genkwa* casbene synthase (Dg*TPS1*)	Increase up to 0.12 mg/g DCW	Du et al. 2023
Casbene	*N. oceanica*	Heterologous co‐expression of *Daphne genkwa* casbene synthase (Dg*TPS1*) and *Coleus forskohlii DXS* with *GGPPS*	Increase up to 1.8 mg/g DCW	Du et al. (2023)
Pentalenene (sesquiterpene)	*C. reinhardtii*	Heterologous expression of codon‐optimised pentalenene synthase (*penA*) from *Streptomyces exfoliatus*	Up to ~10.2‐fold increase	Li et al. (2025)
Isoprene	*C. reinhardtii*	Heterologous co‐expression of *Ipomoea batatas* isoprene synthase (Ib*IspS*) co‐expressed with *Saccharomyces cereviasiae* isopentenyl‐DP delta isomerase yeast (Sc*IDI*)	Increase up to ~10 pg/cell (Ib*IspS*) and up to 19 pg/cell (*IbIspS* + *ScIDI*)	Yahya et al. (2023)
Pinene	*Synechococcus* sp. PCC7002	Heterologous expression of *Abies grandis* pinene synthase (AgPS)	Yields up to 1.525 ± 0.l145 mg/L	Yang et al. (2021)
Geraniol	*P. tricornutum*	Heterologous expression of *Catharanthus roseus* geraniol synthase (Cr*GES*)–episome‐based expression (EE)	Yields up to 0.309 mg/L	Fabris et al. (2020)
		Heterologous expression of *Catharanthus roseus* geraniol synthase (Cr*GES*)–episome‐based expression (EE) vs. randomly integrated chromosomal expression (RICE)	Yield up to 0.15 mg/L (EE) and 0.89 mg/L (RICE)	George et al. (2020)
Bisabolene	*C. reinhardtii*	Heterologous expression of bisabolene synthase from *A. grandis* (Ag*BS*) combined with Cr*SQS* knockdown, amiRNA‐based knockdown of endogenous squalene synthase (Cr*SQS*), and protein farnesyl transferase (Cr*PFT*)	Up to 15‐fold increase	Wichmann et al. (2018)
Patchoulol	*C. reinhardtii*	Heterologous expression of *Pogostemon cablin* patchoulol synthase (Pc*PS*)	Yield up to ~65 μg/L^a^ (2018) and up to ~9 μg/g CDW^a^ (2020)	Baier et al. (2018), (2020)
		Heterologous, combinatorial expression of *Pogostemon cablin* pachoulol synthase (Pc*PS*) combined with amiRNA‐based knockout of endogenous SQS.	Productivity up to 6.2 mg/L (6 days)	Abdallah et al. (2022)
Hemiterpenes (e.g., IPP)	*C. reinhardtii*	Engineered synthetic hemiterpene pathway	Increase up to 8.6‐fold	Zhao et al. (2022)
Isoprenoids (general boost)	*C. reinhardtii*	Full heterologous MVA pathway installation	Enhanced precursor pools; higher terpene titers	Wang et al. (2025)

a Estimated from the graph.

### 4.1 Pathways in Place: MEP, MVA and Their Engineering Potential

**FIGURE 3:**
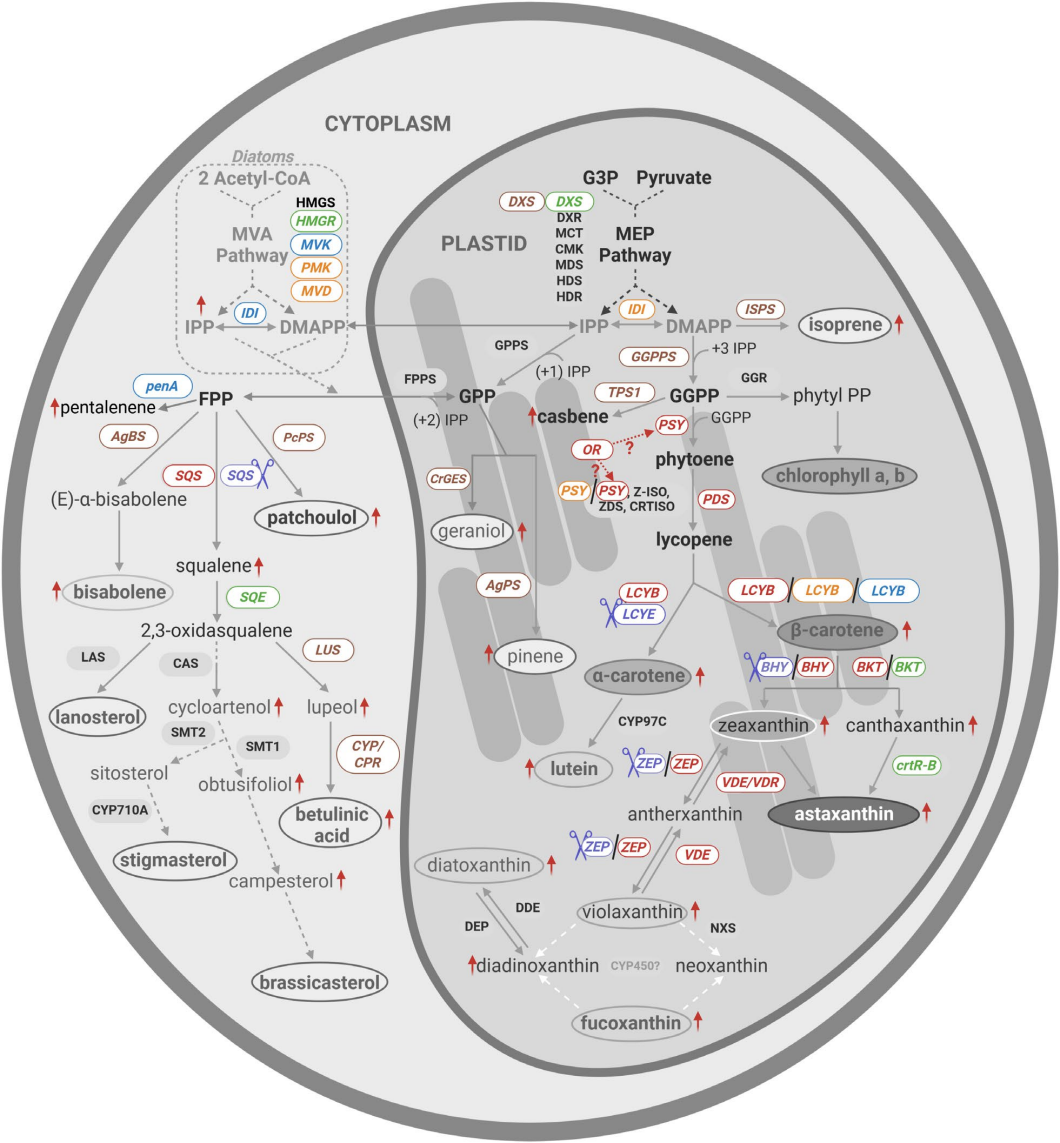
Schematic overview of carotenoid, terpene and sterol biosynthetic pathways in diverse microalgae, highlighting key intermediates and target genes described in Section 4. Genes targeted for endogenous overexpression are shown in red; those disrupted by CRISPR‐Cas9 are in violet; microalgal genes heterologously expressed in other microalgae are in green; bacterial, yeast and plant genes heterologously expressed in microalgae are shown in blue, yellow and brown, respectively. Red arrows indicate increased production of specific compounds (see Tables 3 and 4). Question marks denote the proposed influence of the OR protein on PSY enzymes. Dashed grey arrows represent multistep reactions, while white dashed arrows indicate predicted reactions. Enzyme abbreviations are explained in the main text and in Tables 3 and 4.

The biosynthesis of terpenes and sterols in microalgae has been extensively studied. Thus, here we provide a brief summary emphasising aspects most relevant to the metabolic engineering strategies (Figure 3).

Microalgae synthesise terpenes and sterols through two primary multi‐enzymatic pathways: the mevalonate (MVA) and the methylerythritol phosphate (MEP) pathway (Pu et al. 2021) (Figure 3). While the MEP pathway is plastid‐localised and present in most algal groups, the MVA pathway is restricted to diatoms (Wichmann et al. 2022). The MEP pathway converts pyruvate and glyceraldehyde‐3‐phosphate (G3P) into isopentenyl pyrophosphate (IPP) and dimethylallyl pyrophosphate (DMAPP) via a series of enzymatic reactions, including seven plastidial enzymes (Commault et al. 2019; Jaramillo‐Madrid et al. 2020). In diatoms, the cytosolic MVA pathway begins with the condensation of acetyl‐CoA molecules catalysed by acetyl‐CoA acetyltransferase (ACAT) and 3‐hydroxy‐3‐methylglutaryl‐CoA synthase (HMGS) to form HMG‐CoA, which is reduced to mevalonate by HMG‐CoA reductase (HMGR), the rate‐limiting step (Athanasakoglou and Kampranis 2019). Mevalonate is sequentially phosphorylated and decarboxylated to produce IPP, which is isomerized to DMAPP by isopentenyl diphosphate isomerase (IDI). From IPP and DMAPP, condensation reactions form farnesyl pyrophosphate (FPP) and geranylgeranyl pyrophosphate (GGPP).

FPP serves as a major precursor for sterol biosynthesis, leading to compounds such as lanosterol, stigmasterol, brassicasterol and betulinic acid, while GGPP feeds into carotenoid and terpenoid biosynthesis, including *β*‐carotene, lutein, astaxanthin, patchoulol and geraniol (Fabris et al. 2020; Sathasivam and Ki 2018). Carotenoid biosynthesis starts with the conversion of GGPP to phytoene through phytoene synthase (PSY), followed by desaturation to lycopene, which then branches into α‐carotene and β‐carotene formation through lycopene cyclases (LCYB). Xanthophylls are formed through the hydroxylation of *β*‐carotene by *β*‐carotene hydroxylase (BHY) to form zeaxanthin, which enters the reversible xanthophyll cycle involving violaxanthin de‐epoxidase (VDE) and zeaxanthin epoxidase (ZEP) (Narang et al. 2021). Additional carotenoids such as lutein and astaxanthin are synthesised from *β*‐carotene through sequential hydroxylation and ketolation reactions catalysed by BHY and *β*‐carotene ketolase (BKT), respectively (Saha et al. 2020; Zhang et al. 2020).

Sterol biosynthesis begins with FPP, which is first converted to squalene by squalene synthase (SQS) (Figure 3). In the next step, squalene is oxidised to 2,3‐oxidosqualene, which is then cyclized to form cycloartenol, a key intermediate in the biosynthesis of plant and algal sterols (Fagundes et al. 2020; Gallo et al. 2020). This precursor is then modified via sterol methyltransferases (SMTs), demethylases and cytochrome P450 enzymes into sterols such as campesterol, sitosterol and stigmasterol (Fagundes et al. 2020).

### 4.2 Metabolic Engineering of Microalgae for Enhanced Carotenoid Production

Carotenoids are essential tetraterpenes (C40) in microalgae, where they play crucial roles in light harvesting, energy transfer and protection against photooxidative stress (Sathasivam and Ki 2018). While all photosynthetic organisms synthesise carotenoids, animals must obtain them through diet. In humans, carotenoids are associated with diverse health‐promoting properties, including antioxidant, anticancer, anti‐inflammatory and vision‐supporting functions (Cezare‐Gomes et al. 2019). Metabolic engineering of microalgae to enhance carotenoid production has mainly focused on redirecting carbon flux toward their biosynthesis through modification of both native and heterologous synthases (Figure 3, Table 3).

For example, in *N. oceanica* , overexpression of LCYB led to a 49% increase in *β*‐carotene content (Liu et al. 2022). Further coordinated engineering of carotenoid‐synthesis‐related genes not only boosted *β*‐carotene and lutein, but also affected lipid metabolism in this organism, resulting in higher EPA content (Liu et al. 2024). In turn, in *C. reinhardtii* , expression of a bifunctional phytoene–*β*‐carotene synthase (PBS) from the red yeast *Xanthophyllomyces dendrorhous* increased *β*‐carotene by 38%–72% and lutein by 60%–83% (Rathod et al. 2020). A recent approach in *P. tricornutum* overexpressing *PSY* and *CRTB* homologues also improved *β*‐carotene and fucoxanthin yields (Cen et al. 2022), confirming earlier reports by (Manfellotto et al. 2020), where co‐expression of *VDE*, *VDR* and *ZEP3* also resulted in higher *β*‐carotene content.

As mentioned above, PSY catalyses the first committed and rate‐limiting step in carotenoid biosynthesis (Zhou et al. 2022), while lycopene *β*‐cyclase (LYCB) and carotenoid isomerase (CRTISO) are essential for the efficient formation of *β*‐carotene (Chen et al. 2023). Together, these enzymes represent key targets for metabolic engineering aimed at boosting carotenoid output in both algal and non‐algal hosts. Protein engineering of PSY in *D. salina* also generated transgenic strains with significantly increased β‐carotene accumulation (Liang et al. 2023). Beyond individual enzymes, regulatory elements such as the ORANGE (OR) protein play important roles. OR is believed to stabilise PSY and influence plastid development (Liang et al. 2023), and synergistic regulation between PSY and OR has been shown to further enhance carotenoid accumulation (Zhou et al. 2022). For instance, in *C. reinhardtii* , a single Arg → His substitution in OR increased total carotenoids by 2.8 to 3.7‐fold (Yazdani et al. 2021), establishing OR as a promising target for regulatory and combinatorial engineering with PSY variants.

Downstream engineering of the carotenoid synthesis pathway has been a successful strategy, which enabled the expansion of carotenoid diversity beyond *β*‐carotene. In *C. reinhardtii* , CRISPR/Cas9 knockout of ZEP redirected flux toward zeaxanthin, increasing its yields by ~3‐fold (Jang et al. 2025), while OR overexpression also enhanced violaxanthin levels (Yazdani et al. 2021). Similarly, earlier work using CRISPR‐Cas9 double knockout of ZEP and LCYE generated high‐purity zeaxanthin strains of *C. reinhardtii* , achieving up to 6.84 mg/L zeaxanthin following medium optimisation, with both yield improvement and downstream purification facilitated (Song et al. 2020). In *Synechocystis* sp. PCC6803, introduction of BKT‐ and CRTR‐B‐encoding genes from the freshwater alga *Haematococcus pluvialis* enabled the production of canthaxanthin, adonixanthin and astaxanthin under stress conditions (Liu et al. 2019). A recently tested approach involved introducing the ketolase gene from *C. reinhardtii* (CrBKT) into *N. oceanica* (Canini et al. 2024). This strategy further expanded ketocarotenoid synthesis, with canthaxanthin accumulation successfully demonstrated in *N. oceanica* . Among ketocarotenoids, astaxanthin has received particular attention as a high‐value compound targeted for engineering. In *C. reinhardtii* , expression of a truncated, codon‐optimised BKT resulted in 0.037 mg/g astaxanthin under high light (Perozeni et al. 2020). Rational design of BKT variants combined with a double knockout of LCYC and ZEP in more recent work of the same group further improved astaxanthin yields to 2.8 mg/L, approximately twofold higher than in the abovementioned strains (Perozeni et al. 2025). Recent systematic investigations also identified PSY and CHYB as critical bottlenecks, and their co‐overexpression with BKT achieved up to 23.5 mg/L astaxanthin under high‐cell‐density cultivation, representing a fourfold improvement compared to previous reports in *C. reinhardtii* (Amendola et al. 2023). Beyond this, a CRISPR/Cas9‐mediated knockout of LCYE in *C. reinhardtii* effectively abolished lutein synthesis while redirecting flux to the β‐carotene branch, resulting in a 1.9‐fold increase in zeaxanthin and improved astaxanthin yields when combined with overexpression of *CrBKT*, *PacrtB* and *CrCHYB* (Kneip et al. 2024). In *H. pluvialis* , which naturally accumulates up to 5% astaxanthin by dry weight (Ma et al. 2022), engineering efforts remain comparatively limited. Nevertheless, chloroplast overexpression of phytoene desaturase (PDS) under the psbA promoter resulted in a 1.67‐fold increase in astaxanthin levels (Galarza et al. 2018). Interestingly, diacylglycerol acyltransferase 1 (DGAT1) from *H. pluvialis* , typically associated with triacylglycerol synthesis, was found to catalyse astaxanthin esterification. Moreover, its expression in *E. coli* resulted in enhanced accumulation of astaxanthin (Ma et al. 2022). These findings suggest that co‐overexpression of HpDGAT1 with enzymes from the MEP pathway may further improve both esterification and storage capacity for carotenoids in diverse hosts.

### 4.3 From Isoprene to Casbene: Advances in Non‐Native Terpene Engineering in Microalgae

Significant attention has also turned to the heterologous production of non‐native terpenes in microalgae, including sesquiterpenes, monoterpenes, diterpenes and hemiterpenes. Because algal species do not typically synthesise these compounds, engineering efforts rely on the introduction of plant or microbial terpene synthase genes combined with flux‐boosting strategies within the MEP and MVA pathways. Figure 3 visually represents the metabolic pathways engineered in microalgal cells, while Table 4 summarises the main findings from recent studies, highlighting engineered strains, targeted genes and resulting product yields.

In *C. reinhardtii* , heterologous expression of patchoulol synthase from *Pogostemon cablin* (*PcPS*), coupled with intron‐mediated expression enhancement and SQS knockdown, significantly increased patchoulol yields to ~1.4 mg/L (Abdallah et al. 2022; Baier et al. 2018, 2020). Similar strategies have been applied for bisabolene production in this microalga, where expression of bisabolene synthase from *Abies grandis* (AgBS) combined with FPP flux redirection led to a ~15‐fold improvement (Wichmann et al. 2018). Recent advancements expanded this framework with additional high‐value terpenes. For example, expression of a plant isopentenyl phosphate synthase (IPPS) and phosphatase pair enabled production of hemiterpenes such as isoprene and prenol in *C. reinhardtii* , reaching titers of up to 8.6‐fold higher with respect to the non‐engineered parental strain (Zhao et al. 2022). This represents the first demonstration of efficient hemiterpene biosynthesis in a photosynthetic microorganism. In turn, pentalenene, a valuable sesquiterpene, was produced in *C. reinhardtii* through co‐expression of a codon‐optimised pentalenene synthase (PenS) and metabolic balancing of FPP supply, achieving efficient light‐driven biosynthesis with a 10.2‐fold increase (Li et al. 2025). Using the same host, Yahya et al. (2023) demonstrated that heterologous expression of isoprene synthase (IspS) enabled isoprene titers up to 50–58 mg/L, with co‐expression of auxiliary enzymes such as IDI further boosting yields. Geraniol biosynthesis has also been achieved in diatoms such as *P. tricornutum* through episomal and chromosomal integration of *Catharanthus roseus* geraniol synthase (CrGES), yielding up to 0.89 mg/L (Fabris et al. 2020; George et al. 2020). Monoterpenes such as pinene were similarly produced in *Synechococcus* sp. PCC 7002 using the *A. grandis* pinene synthase (AgPS), reaching ~1.5 mg/L (Yang et al. 2021). At the hemiterpene level, synthetic pathways have been established in algal systems for volatile compounds, such as isopentenol, demonstrating proof of concept for industrial expansion (Zhao et al. 2022).

Additionally, recent work in *N. oceanica* has demonstrated the successful production of casbene, a pharmacologically relevant bicyclic diterpene, through the ectopic expression of the *Daphne genkwa* casbene synthase (DgTPS1), achieving 0.12 mg/g dry cell weight (DCW) (Du et al. 2023). Moreover, co‐expression of this gene with 1‐deoxy‐D‐xylulose 5‐phosphate synthase (DXS), which catalyses the condensation of pyruvate and G3P, and with geranylgeranyl pyrophosphate synthase (GGPPS) from *Coleus forskohlii*, increased casbene production to 1.80 mg/g DCW (Du et al. 2023). Engineering strategies targeting promoter selection have also proven powerful. In *P. tricornutum* , systematic testing of endogenous promoters for the rate‐limiting SQS and HMG‐CoA reductase (HMGR) revealed that promoter strength and regulation strongly influence squalene titres. By selecting a high‐expression promoter, researchers achieved a 5.2‐fold increase in squalene without compromising growth (Nong et al. 2024).

Together, these studies demonstrate that microalgal engineering is moving from single‐enzyme modifications toward system‐level rewiring via complete pathway installation, promoter engineering and combinatorial expression strategies to maximise isoprenoid flux and diversify the terpenoid product spectrum. Collectively, all these works underscore the promise of microalgae as green cell factories for non‐native terpenes, especially when combining terpene synthase expression with flux‐enhancing strategies such as MVA/MEP pathway rewiring, promoter engineering, or intron‐mediated expression. As demonstrated in recent work (Wang et al. 2025), reconstruction of a complete MVA pathway further enhances terpene precursor availability, paving the way for scalable production of non‐native terpenoids in photosynthetic chassis.

### 4.4 Rewiring Sterol Metabolism: Early Steps and Open Questions

In addition to terpenes, sterols are commercially valuable compounds derived from microalgae. While phytosterols occur naturally, efforts have also been made to enhance production through heterologous expression of sterol synthases. Sterol biosynthesis begins with the triterpenoid precursor squalene, which is converted into lanosterol or cycloartenol. These intermediates can then be further transformed into a variety of sterols, including stigmasterol, isofucosterol, fucosterol, campesterol and brassicasterol (D'Adamo et al. 2019; Jaramillo‐Madrid et al. 2020). Despite this commercial potential, metabolic engineering of sterols in microalgae remains at an early stage.

One of the first demonstrations introduced an oxidosqualene cyclase from *Lotus japonicus* into *P. tricornutum* , enabling the synthesis of lupeol at 0.1 mg/g, although this also altered brassicasterol content (D'Adamo et al. 2019). A broader study then examined two rate‐limiting enzymes—3‐hydroxy‐3‐methylglutaryl‐CoA reductase (HMGR) and squalene epoxidase (SQE) in both *P. tricornutum* and *T. pseudonana* . In *P. tricornutum* , HMGR mutants accumulated squalene, cycloartenol, obtusifoliol and 24‐methylcholesta‐5,24(24′)‐dien‐3β‐ol by 10‐fold, 3‐fold, 2.5‐fold and 17‐fold, respectively, compared to wild‐type, while campesterol decreased by half. Similarly, SQE mutants showed 1.8‐fold increases in cycloartenol, obtusifoliol and campesterol. By contrast, *T. pseudonana* HMGR mutants exhibited little overall change, aside from a reduction in fucosterol and isofucosterol. These results suggested that sterol biosynthesis in diatoms is subject to tight metabolic control, likely involving as‐yet‐uncharacterized regulatory mechanisms (Jaramillo‐Madrid et al. 2020). Further work in *P. tricornutum* confirmed this tight regulation but also highlighted opportunities for flux redirection. Overexpression of HMGR or its truncated form (tHMGR) strongly increased squalene (10‐fold and 4‐fold, respectively), along with significant rises in cycloartenol and obtusifoliol. Unexpectedly, these modifications also induced the synthesis of 24‐methylenecholesta‐5,24(24′)‐dien‐3β‐ol, a sterol normally absent in this species, which accumulated up to 17‐fold higher than baseline. At the same time, campesterol levels decreased in HMGR lines but rose threefold in strains expressing a heterologous SQE from *N. oceanica* . Despite these pronounced shifts in intermediates, brassicasterol, the dominant sterol, remained unchanged and total sterol levels were stable, underscoring the strong homeostatic control of sterol pools in *P. tricornutum* . By contrast, *T. pseudonana* proved largely unresponsive to genetic perturbation. Overexpression of its native HMGR, tHMGR, or heterologous NoSQE produced no detectable buildup of intermediates and only minor decreases in fucosterol and isofucosterol. This limited response may reflect inherent differences in sterol regulation between centric ( *T. pseudonana* ) and pennate ( *P. tricornutum* ) diatoms, or constraints imposed by the promoters used (Jaramillo‐Madrid et al. 2020). Together, these studies demonstrate both the potential and the challenges of sterol engineering in diatoms. While *P. tricornutum* shows clear plasticity in intermediate accumulation, global sterol homeostasis is stringently maintained. Achieving substantial and predictable changes in sterol composition will likely require multigene strategies, targeting of downstream branch points, or disruption of regulatory nodes that safeguard sterol balance.

## 5 Conclusions: Perspectives, Emerging Trends and Integrated Strategies

The engineering of PUFAs, carotenoids, terpenes and sterols in microalgae illustrates a clear transition from early single‐enzyme modifications toward integrated, system‐level rewiring strategies. The recent progress reviewed here indicates that meaningful advances now depend on combining multiple approaches—heterologous gene expression, promoter optimisation, protein engineering, CRISPR‐mediated editing and full pathway reconstruction. For instance, carotenoid engineering has successfully coupled enzyme optimisation with regulatory protein manipulation and storage pathway design, enabling both higher yields and diversification of products such as zeaxanthin and astaxanthin. Similarly, terpene production has been expanded through co‐expression of terpene synthases with upstream flux‐enhancing enzymes, leading to measurable yields of sesquiterpenes, monoterpenes and hemiterpenes. In sterols, targeted interventions have revealed both the robustness of homeostatic control and new opportunities for pathway redirection. Collectively, these studies converge on the emerging consensus that microalgae can serve as versatile green cell factories for complex isoprenoids, provided engineering strategies embrace integrated, combinatorial and system‐level designs.

To fully harness this potential, metabolic engineering must focus on more efficient redirection of carbon flux toward PUFA, terpene and sterol pathways. Strategies such as overexpressing bottleneck enzymes, eliminating competing reactions, and sourcing highly efficient enzymes from diverse species are critical. Here, the convergence of empirical validation with advanced computational tools, particularly enzyme structure prediction via platforms such as *PyMol* and *AlphaFold*, offers powerful avenues for rational protein engineering and synthetic pathway design.

Integrating these data into curated metabolic databases will accelerate pathway optimisation and flux balancing. As such resources mature, the overexpression of complete pathways (e.g., MEP or PUFA elongase/desaturase routes) within optimised microalgal strains will become increasingly feasible. Genome‐scale models informed by proteomics and enzyme kinetics will further improve the predictive accuracy of metabolic simulations, enhancing strain design success rates. Yet, regulation at transcriptional, post‐transcriptional and epigenetic levels remains a key bottleneck. The identification of key transcription factors, safe harbour loci and enhancers, coupled with the adoption of single‐cell transcriptomics, promises to shed light on cellular heterogeneity and fine‐scale regulatory mechanisms. Moreover, understanding the influence of DNA methylation, histone modifications and non‐coding RNAs may be critical to overcoming silencing effects and stabilising production.

Another recurring challenge lies in mitigating cellular toxicity caused by the overaccumulation of lipids and terpenoids. While volatile terpenes can be extracted using overlays such as dodecane, PUFAs and sterols are membrane‐associated or lipid droplet (LD)‐bound, often disrupting cell physiology. Engineering LDs biogenesis, through the co‐expression of LD‐surface proteins, Kennedy pathway components and PUFA‐synthesising enzymes, provides a promising solution for the safe intracellular storage of these compounds.

To summarise, we propose the following conceptual roadmap for microalgal metabolic engineering: (1) establish comprehensive genome‐scale models with integrated proteomics; (2) identify robust, species‐specific expression systems and safe harbour loci; (3) engineer high‐efficiency multigene constructs using modular cloning systems; (4) integrate feedback‐driven learning via the DBTL framework and (5) apply these tools across both model and extremophilic algal strains. By addressing these milestones, the microalgal research community can accelerate progress toward economically viable production of PUFAs, terpenes and sterols.

## Conflicts of Interest

The authors declare no conflicts of interest.

## Data Availability

Data sharing not applicable to this article as no datasets were generated or analysed during the current study.
